# Orthographic and feature-level contributions to letter identification

**DOI:** 10.1177/17470218221106155

**Published:** 2022-06-28

**Authors:** Clare Lally, Kathleen Rastle

**Affiliations:** 1 Royal Holloway, University of London, London, UK; 2 UCL Speech, Hearing and Phonetic Sciences, University College London, London, UK

**Keywords:** Visual word recognition, reading, letter identification, visual processing, orthographic processing

## Abstract

Word recognition is facilitated by primes containing visually similar letters (*dentjst-dentist*), suggesting that letter identities are encoded with initial uncertainty. Orthographic knowledge also guides letter identification, as readers are more accurate at identifying letters in words compared with pseudowords. We investigated how high-level orthographic knowledge and low-level visual feature analysis operate in combination during letter identification. We conducted a Reicher–Wheeler task to compare readers’ ability to discriminate between visually similar and dissimilar letters across different orthographic contexts (words, pseudowords, and consonant strings). Orthographic context and visual similarity had independent effects on letter identification, and there was no interaction between these factors. The magnitude of these effects indicated that high-level orthographic information plays a greater role than low-level visual feature information in letter identification. We propose that readers use orthographic knowledge to refine potential letter candidates while visual feature information is accumulated. This combination of high-level knowledge and low-level feature analysis may be essential in permitting the flexibility required to identify visual variations of the same letter (e.g., N-n) while maintaining enough precision to tell visually similar letters apart (e.g., n-h). These results provide new insights on the integration of visual and linguistic information and highlight the need for greater integration between models of reading and visual processing.

## Introduction

Understanding the processes that underpin letter identification has been a long-standing goal within experimental psychology. Readers must maintain enough flexibility to recognise that *gate* and *GATE* are the same word, but also enough precision to recognise that *gate* and *gale* are not. Research shows that readers activate letter representations rapidly despite wide-ranging variability in their visual form (e.g., case and font; Bowers et al., 1998; Hannagan et al., 2012; Kinoshita & Kaplan, 2008). However, existing literature also reveals that this flexibility extends beyond letter identity in the initial moments of visual word recognition. Masked priming paradigms demonstrate that word recognition is facilitated by prior presentation of stimuli that contain visually similar letters (*dentjst-DENTIST* vs *dentgst-DENTIST*, Marcet & Perea, 2017; *docurnent–DOCUMENT* vs *docusnent–DOCUMENT*; Marcet & Perea, 2018a), numbers (*C4BLE-cable* vs *C9BLE-cable;*Kinoshita et al., 2013; Perea et al., 2008), or symbols (*C^BLE-CABLE;*Perea et al., 2008). Evidence from eye-tracking shows facilitation from visual feature similarities, shown by shorter fixation times for target words in sentences when parafoveal preview contains a pseudoword neighbour with a visually similar letter compared with a visually dissimilar letter (e.g., *frjed-fried* vs *frged-fried*; Marcet & Perea, 2018b). Event-related potential data also demonstrate that strings containing letter-like numbers can facilitate lexical access, as such strings evoke similar N400 semantic responses to the words they resemble (*4PPL3-APPLE;*Lien et al., 2014). Together, these findings suggest that the process of letter identification may consist of an accumulation of information about visual features.

Readers draw upon their knowledge of the writing system to support letter identification processes. For example, readers adjust prioritisation of different visual features as they gain expertise in an unfamiliar alphabet, to discriminate between letters (Wiley et al., 2016). Letter identification is guided by orthographic knowledge, such as knowledge of legal letter combinations or existing words. Consequently, the contexts in which letters appear can significantly alter readers’ ability to discriminate between them. Readers identify letters more accurately when they appear in a real word compared with a pseudoword (Coch & Mitra, 2010; Grainger & Jacobs, 1994; Kezilas et al., 2016; Reicher, 1969; Wheeler, 1970). This *word superiority effect* is understood as evidence that word representations enrich letter identification processes (Grainger & Jacobs, 1996; McClelland & Rumelhart, 1981; Rumelhart & McClelland, 1982). Letter identification is also more accurate in pronounceable pseudowords (*pable*) compared with unpronounceable consonant strings (*pkwtj*) (Baron & Thurston, 1973; Carr et al., 1978). This *pseudoword superiority effect* suggests that letter identification is also guided by readers’ knowledge of orthotactic constraints (i.e., restrictions on how letters combine within a writing system; Kezilas et al., 2016). Thus, orthographic knowledge appears to play a key role in resolving early uncertainty around letter identity, and may reduce confusability from shared letter features. However, this line of research has not generally tested or controlled for effects of visual feature similarity.

Other work has explored whether precise visual feature information is less influential on letter identification when top-down orthographic information is available to compensate. Researchers have investigated this question by distorting the visual appearance of letters and measuring readers’ abilities to recognise them in different letter string contexts. Letter distortion is more disruptive in single letters (Fiset et al., 2008) and pseudowords (Rosa et al., 2016) compared with real words. Therefore, existing research indicates that readers use orthographic knowledge to resolve inconsistencies in visual feature information, whether it is distortion from visual noise (Fiset et al., 2008; Rosa et al., 2016) or substitution of a visually similar letter appearing in a word-like string (e.g., *dentjst*, Marcet & Perea, 2017). However, these scenarios typically involve readers encountering an invalid string and measuring how quickly they recognise the closest word neighbour. Less is known about whether orthographic context reduces ambiguity from visual feature similarity if both letters result in an equally valid string. Readers often encounter this situation, as they must distinguish between word neighbours with similar looking letters (e.g., *gate-gale*). Based on previous findings, we would expect visually similar neighbours (*gate-gale*) to be harder to distinguish than visually dissimilar neighbours (*gate-game*). But how does letter confusability change across orthographic contexts? To our knowledge, researchers are yet to investigate whether orthographic context mediates readers’ ability to discriminate between visually similar letters if they both result in a string with an equivalent word or non-word status.

High-level orthographic knowledge and low-level visual feature analysis both play a key role in letter identification, but less is known about how they interact. The visual forms of letters are highly variable; therefore, readers may use orthographic context to compensate for inconsistencies in visual feature information. Orthographic distributional knowledge provides information on how individual characters relate to each other, as readers can learn from the contexts in which letters co-occur (Schubert et al., 2020). This knowledge can reinforce mappings between variable letter shapes and identities, provide cues on the expected visual form (such as case and font), and assist in refining potential letter candidates while visual feature information is still being accumulated. These context cues not only assist readers in overcoming within-letter visual variability but also reduce the likelihood of confusing visually similar letters. Therefore, cues from orthographic context may play a role in constraining letter candidates to manage the balance of flexibility and precision required during letter identification. If so, letter confusability from visual similarity may be reduced when wider orthographic information is available.

The focus of this work was to examine how high-level orthographic knowledge and low-level visual feature analysis work in tandem during letter identification. We conducted a Reicher–Wheeler task to compare readers’ abilities to discriminate between letters with high and low visual feature similarity across words, pseudowords, and consonant strings. We predicted that readers would be less accurate at discriminating between two letters with high visual feature overlap (*m-n*) relative to two letters with low visual overlap (*m-t*). We also predicted that letter identification would be more accurate in words relative to pseudowords, and pseudowords relative to unpronounceable consonant strings, in line with word (Reicher, 1969; Wheeler, 1970) and pseudoword superiority effects (Baron & Thurston, 1973; Carr et al., 1978). Finally, we predicted that letter confusability from visual similarity would be reduced when letter-strings aligned with orthographic and orthotactic knowledge, as we proposed that readers would use their knowledge of words and legal letter combinations to narrow down plausible letter candidates. Therefore, we predicted an interaction where accuracy differences between letters with high and low visual feature similarity would be smaller in words compared with pseudowords, and in pseudowords compared with consonant strings.

## Method

### Data availability

This study was pre-registered on the Open Science Framework (OSF). Pre-registration, stimuli, instructions, trial-level data, and analysis scripts are openly available (https://osf.io/p4q9u/).

### Participants

Seventy-two monolingual English speakers completed the experiment at Royal Holloway University of London, in exchange for £5. All participants were aged 18–35, with normal or corrected-to-normal vision, and no previous history of reading difficulty. The sample size was determined alongside the number of items (24 items per condition) to meet the suggested criterion of 1,600 observations per condition for analyses using linear mixed-effects models (24 × 72 = 1,728 observations per condition, Brysbaert & Stevens, 2018). All participants provided informed consent prior to taking part.

### Stimuli

Target stimuli consisted of 48 words, 48 pronounceable pseudowords, and 48 unpronounceable consonant strings. These three target stimuli conditions comprised the independent variable of orthographic context. Each target stimulus was assigned a target letter that was present within the stimulus, and two possible foil letters that were not present in the stimulus. Foil letters had either high visual feature overlap or low visual feature overlap with the target letter. This manipulation formed our second independent variable: visual feature similarity (high vs low). The critical target and foil letters included in visual similarity comparisons were the same across each orthographic context condition. Substitution of the target letter with either of the foil letters always resulted in a string with the same orthographic context status as the target (e.g., word: *snow/show/stow*, pseudoword: *snum/shum/stum*, consonant string: *znsq/zhsq/ztsq*). All letter strings were four to six letters long, and words and pseudowords had a single-syllable pronunciation. Word targets (*snow*) and words with the substituted foil letter (*show/stow*) were controlled for frequency using the CELEX database (Baayen et al., 1995). Stimuli for a preliminary staircase-thresholding task consisted of an additional 20 words, 20 pseudowords, and 20 consonant strings, with the same control measures as those used for the main task. None of the stimuli presented in the thresholding task were present in the main task.

Visual feature similarity was quantified using 7-point letter similarity ratings from over 700 people (Simpson et al., 2013). Target letters had a mean similarity rating of 4.19 with foil letters in the high-overlap condition compared with 1.22 with foil letters in the low-overlap condition, *t*(47) = 24.8, *p* < .001. This difference between high- and low-overlap conditions was confirmed with a second, objective measure of visual similarity derived from the Hierarchical Model and X (HMAX, Mutch & Lowe, 2008), a biologically motivated computational model that mimics properties of the human ventral visual system through a series of simple (S1, S2) and complex (C1, C2) layers. We used HMAX S1 layer computations to calculate letter similarities, as this layer was modelled upon the earliest instance of feature detection. HMAX calculations revealed that target letters had a mean similarity rating of 0.59 with foil letters in the high-overlap condition compared with 0.50 with foil letters in the low-overlap condition, *t*(47) = 6.25, *p* < .001. HMAX and reader ratings were positively correlated, *r*(323) = .49, *p* < .001.

### Procedure

Participants completed a Reicher–Wheeler task consisting of 144 trials, administered using DMDX (Forster & Forster, 2003). Within each trial, participants viewed a 500 ms fixation cross, followed by a forward mask for 33 ms. A target letter-string (a word, pseudoword, or consonant string) then appeared for a predetermined duration (see below), before a hash symbol (#) backward-masked each letter of the target for 100 ms. During this time, a probe bar (|) appeared above and below one of the hash symbols, which indicated that the participant should identify the letter in the specified position. After 100 ms, a target letter and a foil letter replaced the probe bars above and below one of the hash symbols. The foil letter had either high visual feature similarity or low visual feature similarity to the target letter. Participants then had 5,000 ms to make a button-press response to indicate which of the two letters was present within the string. Targets were counter-balanced to ensure that participants received an equal number of foil letters across high and low visual feature similarity conditions and to ensure that participants saw each target letter-string once.

Target exposure duration was determined for each participant based on performance in an initial staircase-thresholding task (adapted from Davis, 2001), which used the same trial procedure and mask durations as the main task. In the thresholding task, exposure duration began at 33 ms and adjusted after each response. If the participant correctly identified the target letter, exposure duration was reduced by 17 ms. If the participant incorrectly identified the foil letter, exposure duration increased by 17 ms. Exposure duration was held constant after 12 changes in direction, and this value set target exposure duration for each participant in the main task. Exposure during the main experiment was 33 ms for 36 participants, 50 ms for 22 participants, 67 ms for 13 participants, and 83 ms for 1 participant. The exposure durations were similar to previous Reicher–Wheeler studies with skilled adult readers (Chase & Tallal, 1990; Coch & Mitra, 2010; Grainger et al., 2003; Kezilas et al., 2016; Lété & Ducrot, 2008).

## Results

**Figure 1.**
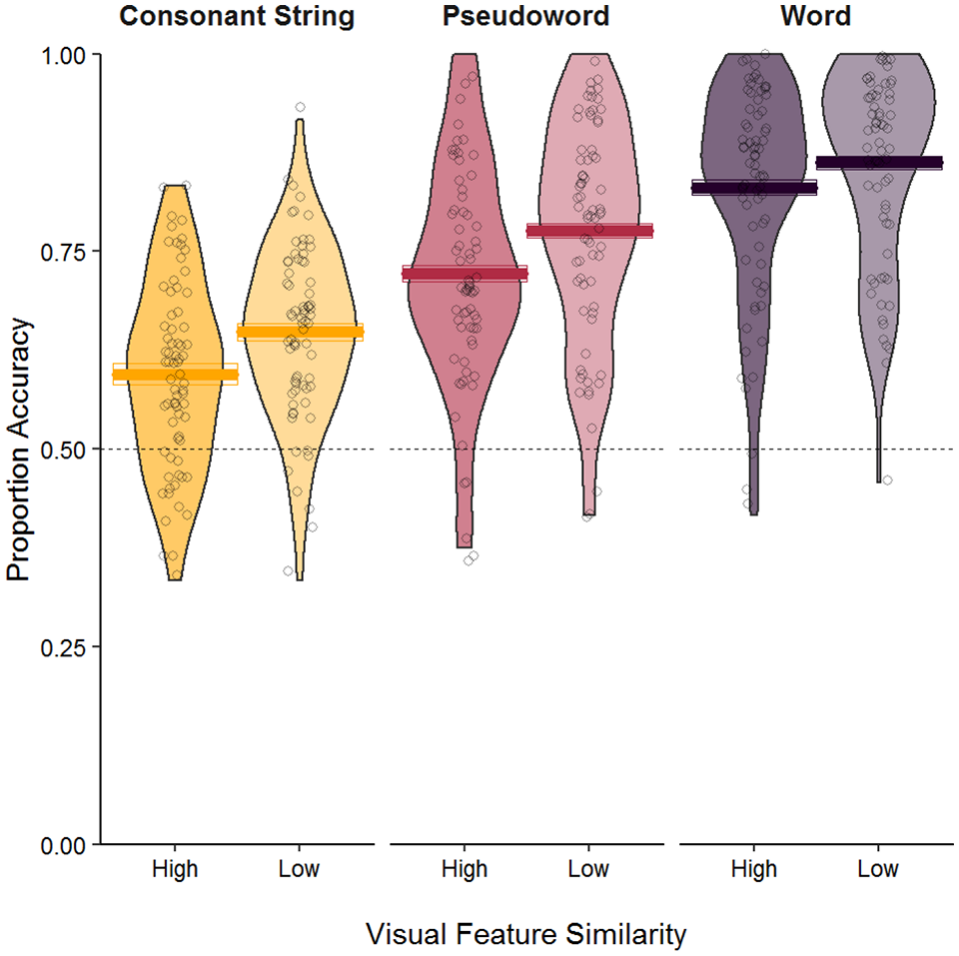
Mean accuracy rates for letter identification in the Reicher–Wheeler task. Crossbars display mean accuracy rates across participants and tiles display one standard error from the mean, calculated for within-subject designs (Loftus & Masson, 1994). Data points display accuracy rates for individual participants and violins demonstrate the distribution of the data. The dashed horizontal line displays chance performance.

Mean accuracy results are visualised in Figure 1. Accuracy data were analysed using logistic generalised linear mixed-effects models with the *lme4* package (Version 1.1-12; Bates et al., 2015) in R (Version 4.0.4; R Core Team, 2016). The maximal model was defined as: glmer (Accuracy ~ Exposure Duration + (Visual Feature Similarity × Orthographic Context) + (1|Participant) + (1|Item), family = binomial). Continuous predictors (exposure duration) were centred around the mean. Categorical factor predictors (visual feature similarity and orthographic context) were dummy coded, which resulted in each level of the factor being compared to a specific level acting as a reference. For the fixed effect of visual similarity, accuracy in the high visual similarity condition was compared to accuracy in the low visual similarity condition as the reference. For orthographic context, accuracy performance for words and pseudowords was compared to accuracy performance for consonant strings as the reference. Therefore, the intercept of the model referred to accuracy performance within the two reference conditions (discriminating between low visual similarity letters in consonant strings).

We investigated whether each component improved the model fit using pairwise likelihood ratio tests (LRTs), in which random effects, main effects, and the interaction term were systematically added in turn (Matuschek et al., 2017). The model fit was improved by random effects of participant, LRT: χ^2^(1) = 269.76, *p* < .001, and item, LRT: χ^2^(1) = 160.59, *p* < .001. We then added the fixed effect of exposure duration, which referred to the duration each letter string was presented for based on participant performance in the preliminary thresholding task. The fixed effect of duration exposure continued to improve the fit of the model, LRT: χ^2^(1) = 16.75, *p* < .001. Next, we included our fixed effects of interest. The fit of the model significantly improved after including the fixed effects of orthographic context, LRT: χ^2^(2) = 494.61, *p* < .001, and visual feature similarity, LRT: χ^2^(1) = 32.24, *p* < .001. However, including the interaction term did not significantly improve the model fit, LRT: χ^2^(2) = 0.36, *p* = .838. This indicated that there was no significant interaction between visual feature similarity and orthographic context. We opted to preserve the interaction term despite it not improving the fit, as this enabled us to test our pre-established confirmatory hypothesis that orthographic context mediates effects of visual feature similarity (see Roettger, 2019). After establishing the model fit, we ran the model and iteratively redefined the dummy-coded reference level of orthographic context to systematically compare all levels to each other. Fixed and random effects results are reported in Table 1. Beta (β) and odds ratios (ORs) are used to report effect sizes. β is the logit transformed fixed effect coefficient, which refers to the estimated difference between conditions having controlled for random effects. OR (derived from β) measures the difference in odds of being correct (vs incorrect) in one level of a fixed effect compared to another.

**Table 1.** Logistic generalised linear mixed-effects model output for analysis of exposure duration, visual feature similarity, and orthographic context on letter identification.

Fixed effects	β (SE)	OR (SE)	*z*	*p*
Exposure duration	0.02 (0.00)	1.02 (0.00)	4.31	<.001
Visual Feature Similarity: High vs Low	–0.25 (0.07)	0.78 (0.06)	–3.37	<.001
Orthographic Context: Pseudoword vs Consonant String	0.68 (0.08)	1.98 (0.16)	8.62	<.001
Orthographic Context: Word vs Consonant String	1.31 (0.09)	3.70 (0.33)	14.74	<.001
Orthographic Context: Word vs Pseudoword	0.62 (0.09)	1.86 (0.17)	6.70	<.001
Visual Similarity × Orthographic Context: Pseudoword vs Consonant String	–0.06 (0.11)	0.94 (0.10)	–0.57	.569
Visual Similarity × Orthographic Context: Word vs Consonant String	–0.01 (0.12)	0.99 (0.12)	–0.07	.946
Visual Similarity × Orthographic Context: Pseudoword vs Word	–0.05 (0.13)	0.95 (0.12)	–0.43	.669
Random effects				
σ^2^	3.29		τ00	0.19 _SubID_
ICC	0.09			0.15 _Item_
Observations	10,368		*N*	72 _SubID_
Marginal *R*^2^/Conditional *R*^2^	.091/.176			48 _Item_

SE: standard error; OR: odds ratio; ICC: intraclass correlation coefficient.

Beta and odds ratio effect sizes are reported, with standard error in parentheses.

There was a significant main effect of visual feature similarity on letter identification accuracy. The odds ratios suggest that participants were 0.78 times as likely (or 22% less likely) to select the correct letter when the foil letter had high visual overlap with the target. There was also a significant main effect of orthographic context on letter identification accuracy. Participants were 1.86 times more likely to correctly identify the target letter in words relative to pseudowords and 3.70 times more likely to correctly identify the letter in words relative to consonant strings. Participants were also 1.98 times more likely to correctly identify the letter in pseudowords relative to consonant strings.

There was no evidence of an interaction between visual feature similarity and orthographic context. The interaction term did not significantly improve the fit of the model and the estimated odds ratios for interaction effects were close to 1 (ORs: 0.94–0.99 or between 1% and 6% less likely), which indicates an equivalent likelihood of high visual similarity reducing letter identification accuracy in either orthographic context. These estimated reductions are unlikely to predict a meaningful difference, as the degree of uncertainty (shown by standard error in Table 1) indicates that each of these estimates could span either side of OR = 1 with sampling error considered. As an additional measure, we conducted exploratory Bayesian analyses to establish whether the absence of an interaction provided evidence for the null hypothesis (i.e., that effects of visual feature similarity are not modulated by orthographic context), or whether there was insufficient evidence to infer a conclusive outcome. Using the motivated maximum approach (Silvey et al., 2021, based on principles of Dienes, 2014), we calculated Bayes factors (BFs) from the interaction estimates produced by our logistic mixed-effects model. BFs were calculated using a half normal distribution (HN). Bayesian results are reported with parentheses expressing the mode of the distribution (first number), and the standard deviation (second number) in convention with Silvey et al. (2021). All BFs were lower than 0.3 (Visual Similarity × Pseudoword vs Consonant String: BF_HN(0,0.11)_ = 0.11, Visual Similarity × Word vs. Consonant String: BF_HN(0,0.12)_ = 0.17, Visual Similarity × Word vs. Pseudoword: BF_HN(0,0.13)_ = 0.05). This indicated moderate evidence for the null hypothesis (Lee & Wagenmakers, 2014; Silvey et al., 2021) that orthographic context does not mediate effects of visual similarity.

Our Bayesian analyses provided evidence that there was no interaction. However, there remains a small possibility that our study was underpowered to detect it (Brysbaert, 2019), despite our relatively large sample size (*N* = 72) and large number of observations per condition (Brysbaert & Stevens, 2018). Thus, we ran Monte Carlo power analyses on simulated datasets to estimate the interaction effect sizes that could have been reliably detected with our sample size. Power analyses and measures taken to protect against issues of post hoc interpretation are reported in further detail on the OSF. Using the *simr* package (Version 1.0.5; Green & MacLeod, 2016) in R (Version 4.0.4; R Core Team, 2016), we systematically increased hypothetical interaction effect sizes by β = 0.05 and ran 50 simulations for each increment, beginning at β = 0.1. For each simulation, we modelled a larger effect between words and consonant strings relative to words and pseudowords under our hypothesis that visual similarity effects would have a greater impact on letter identification when less orthographic information is available. Our sample size (*N* = 72) yielded 80% power to detect an interaction with an effect size of β = 0.3 between visual similarity differences in words and pseudowords, and an effect size of β = 0.4 between visual similarity differences in words and consonant strings. The equivalent odds ratios demonstrate that we had the power to detect an interaction if the benefit of having two visually distinct letters was at least 1.35 times more likely to improve letter discrimination in pseudowords relative to words, and 1.49 times more likely in consonant strings relative to words. These analyses show that, if there was an undetected interaction between visual similarity and orthographic context in our data, it was smaller than the effect sizes stated above.

## Discussion

Our results revealed effects of orthographic context and visual feature similarity on letter discrimination accuracy in a Reicher–Wheeler task. Performance improved as letter strings became more word-like (words > pseudowords > consonant strings), replicating the word superiority effect and the pseudoword superiority effect (Baron & Thurston, 1973; Carr et al., 1978; Reicher, 1969; Wheeler, 1970). Performance was also superior when the discrimination involved letters with low visual similarity compared with letters with high visual similarity. There was no interaction between the effects of orthographic context and visual feature similarity; visually similar letters were more confusable irrespective of how closely the target letter string aligned with a real word. Odds ratios indicated that effects of orthographic context were much larger than effects of visual feature similarity, which suggests that top-down orthographic knowledge may be relatively more important than bottom-up feature information in establishing letter identities.

We had hypothesised that there would be an interaction between visual similarity and orthographic context, such that effects of visual feature similarity would be stronger where there is less high-level orthographic information available. However, there was no evidence to suggest that the impact of visual similarity on letter confusability varied across word, pseudowords, or consonant strings. Exploratory Bayesian analyses indicated moderate evidence against an interaction, although increased power could provide the benefit of greater certainty. Power simulations revealed that our design was not powered to detect the interaction effect sizes revealed in the model output (Table 1); thus, these estimates of the interaction effect size need to be treated with caution. However, detecting an interaction effect of this size would require a sample of at least 5,000 participants (based on 80% power, calculations available on the OSF). It may be more constructive in future research to determine what an ecologically meaningful interaction effect size would be and calculate power accordingly.

Our findings advance current understanding of letter identification in several ways. First, we believe this work to be one of the first to demonstrate effects of visual feature similarity on letter identification within a Reicher–Wheeler paradigm. This departs from previous work investigating visual feature similarity, which has mostly been restricted to masked priming (although see also Marcet & Perea, 2018b, for parafoveal preview effects). The current work demonstrates that effects of visual feature similarity are not task-specific and have implications for multiple levels of processing. Pre-existing evidence from masked priming demonstrated that visual feature similarity has a discernible influence on low-level perceptual processing (i.e., visual similarity between the prime and the target) and processes that rely upon broad lexical knowledge (i.e., visual similarity between the prime and known word strings), whereas the current findings show that visual feature similarity also impacts processing when readers are required to specifically discriminate between letter candidates.

Second, this study has taken a new approach to investigating visual similarity effects, by investigating the impact of shared featural information across letters that result in equally valid letter strings (i.e., both letters result in a real word, for example). In masked-priming paradigms, researchers have typically compared visual overlap between pseudoword and word neighbours, whereby the pseudoword is the prime and the word neighbour is the target (e.g., *dentjst-dentist* vs *dentgst-dentist*; Marcet & Perea, 2017). This has yielded powerful initial evidence that shared featural information can be beneficial for visual word recognition, as readers are faster at overcoming discrepancies between a letter string and the closest known word form if the two are visually similar. When relating to real-life reading experience, this advantage is akin to recovering from a typing or spelling mistake. In contrast, the current work investigates an alternative problem, as it is one of the first to investigate the influence of visual similarity when discriminating between letters that result in equally plausible word forms. In this scenario, readers are not assessing overlap between the visual input and an expected letter form, but instead distinguishing between competing letter identities. This is an alternative but also common challenge during reading, as readers routinely discriminate between word neighbours with similar looking letters (e.g., *gate-gale*). This critical difference provided a new insight: visual feature similarity may benefit visual word recognition when one letter is more likely to occur than the other, but it can also be disadvantageous if both letters are equally plausible, as it hinders readers’ ability to discriminate between them. This suggests that readers refine potential letter candidates while visual feature information is still being accumulated. Visual feature similarity impedes visual word recognition when both letters are equally viable, as neither competitor has been disregarded as an unsuitable candidate.

This interpretation is further supported by an additional novel conclusion from this work, which relates to how cues are weighted during letter identification. The influence of orthographic context was much larger than the influence of visual feature similarity, which suggests that top-down orthographic knowledge may be prioritised over bottom-up feature information during letter identification. This differential weighting may again occur because orthographic knowledge plays a critical role in filtering letter candidates, enabling readers to maintain the balance of flexibility and precision required for letter identification. Readers must incorporate a certain degree of flexibility when mapping low-level visual features to letter identities, as the visual appearance of letters can be highly variable. However, allowing greater flexibility also increases the risk of letter confusability. We propose that orthographic knowledge mitigates this risk while visual feature information is still being accumulated, by disregarding unlikely letter candidates and prioritising those that would result in a real word or an orthotactically legal letter string.

This finding has important theoretical implications, as understanding the weighting attributed to word-level (or “string-level”) cues relative to visual feature cues is essential for understanding the integration of visual and linguistic information. This proposed “mid-level vision stage” of orthographic processing (Grainger, 2018) is often under-specified in cognitive models of reading, as feature-level processes are either assumed a priori or minimally outlined (Marcet & Perea, 2017). There has been greater focus in neuro-biological models of reading. These models propose that mechanisms for visual object identification interact with linguistic processes to facilitate visual word recognition. For example, the local combination detector model (Dehaene et al., 2005) proposes that readers hierarchically encode increasingly large fragments of orthographic information that advance in linguistic complexity (features, letter shapes, abstract letter identities, bigrams, substrings) in the visual ventral stream, with increasing sensitivity to linguistic probabilities within the writing system. The model outlines how feature-level information may be incorporated based on principles of the primate visual system; readers amalgamate oriented bars and local contours to detect letter shapes, which are then used to inform abstract letter representations invariant of font or case. Thus, the local combination detector model is able to explain why visually similar letters are more confusable. However, this model assumes a one-way feed-forward approach, which restricts its ability to explain how orthographic knowledge reduces letter confusability. Without incorporating feedback, it is difficult to align this account with our finding that orthographic status has a much larger influence on letter confusability than low-level visual similarity, particularly as this was observed in a Reicher–Wheeler task with limited exposure to the visual input.

Alternatively, the influence of orthographic knowledge on refining letter candidates can be characterised by principles of cascaded processing, whereby later stages of word processing are implemented before earlier stages are completed (McClelland, 1979). Cascaded processing can explain why effects of visual feature similarity are outweighed by cues from lexical information when available, as word-level feedback plays a greater role in activating letter representations compared to bottom-up activation from feature-level information alone. Recent neural evidence has indicated how cascaded processing may be incorporated into existing neuro-biological models, following detection of feedforward and feedback activity within the ventral stream (Woolnough et al., 2020). Woolnough et al. (2020) found that posterior regions were the earliest to show increased activation in response to orthographic stimuli; however, these regions also demonstrated sensitivity to lexical status later than anterior regions. Differences in early and late selectivity could reflect cascaded processing, as word-likeness recognised in anterior regions may propagate backwards and interact with letter-level processing in posterior regions. Thus, there is potential to inform a cascaded model which could incorporate both the analysis of visual information and feedback from linguistic knowledge.

The greater weighting attributed to high-level orthographic information could otherwise potentially be explained by Bayesian models of reading, which propose that visual word recognition is achieved by readers combining tentative evidence with knowledge of prior probability (Norris, 2006; Norris & Kinoshita, 2012; Norris et al., 2010). Under this interpretation, bottom-up analysis of low-level orthographic features constitutes the tentative evidence and integration of top-down orthographic knowledge shapes the priors of the expected visual word representation. The greater influences of high-level orthographic cues (i.e., word status) relative to low-level visual cues (i.e., feature information) may be due to readers having stronger priors for letter combinations associated with known word representations or phonotactically legal letter combinations, which requires less detailed analysis of the visual evidence. It is less clear how these principles would be ingrained in visual processing, although there is again potential to consider the compatibility of these principles with existing neuro-biological or visual models of reading. For example, previous neuro-imaging work has documented how top-down predictions influence the sensory processing of speech (Davis & Sohoglu, 2020; Sohoglu et al., 2012). Future work could investigate similar principles for reading within the visual domain.

In conclusion, the current study demonstrated that letter identification is supported through a balance of information from visual features and high-level orthographic knowledge. Our results showed that visually similar letters are more confusable than dissimilar letters, indicating that readers encode letter identities with initial uncertainty, based on feature information. Word and pseudoword superiority effects demonstrated that readers also use orthographic knowledge of known words and legal letter combinations to resolve early uncertainty around letter identity. In the absence of an interaction, there is no evidence to suggest that orthographic context mediates effects of visual similarity specifically. Instead, our findings indicate that orthographic knowledge and visual feature similarity have an additive effect on letter identification. This work provides a novel insight that high-level orthographic information plays a greater role than low-level visual feature information in letter identification. We suggest that this is a result of readers using orthographic knowledge to refine potential letter candidates efficiently while visual feature information is still being accumulated. More broadly, this work advances understanding of the integration of visual and linguistic information and highlights the need for greater cross-examination between models of reading and visual processing.

## References

[bibr1-17470218221106155] Baayen R. H. Piepenbrock R. van Rijn H. (1995). The CELEX Lexical Database. Release 2 [CD-ROM]. Linguistic Data Consortium, University of Pennsylvania, Philadelphia, PA.

[bibr2-17470218221106155] Baron J. Thurston I. (1973). An analysis of the word-superiority effect. Cognitive Psychology, 4(2), 207–228. 10.1016/0010-0285(73)90012-1

[bibr3-17470218221106155] Bates D. Maechler M. Bolker B. Walker S. (2015). lme4: Linear mixed-effects models using Eigen and S4 (R Package Version 1.1-7. 2014).

[bibr4-17470218221106155] Bates D. Mächler M. Bolker B. Walker S. (2015). Fitting linear mixed-effects models using lme4. Journal of Statistical Software, 67(1), 1–48. 10.18637/jss.v067.i01

[bibr5-17470218221106155] Bowers J. S. Vigliocco G. Haan R. (1998). Orthographic, phonological, and articulatory contributions to masked letter and word priming. Journal of Experimental Psychology: Human Perception and Performance, 24(6), 1705–1719. 10.1037/0096-1523.24.6.1705 9861718

[bibr6-17470218221106155] Brysbaert M. (2019). How many participants do we have to include in properly powered experiments? A tutorial of power analysis with reference tables. Journal of Cognition, 2(1), Article 16. 10.5334/joc.72 PMC6640316 31517234

[bibr7-17470218221106155] Brysbaert M. Stevens M. (2018). Power analysis and effect size in mixed effects models: A tutorial. Journal of Cognition, 1(1), Article 9. 10.5334/joc.10 PMC6646942 31517183

[bibr8-17470218221106155] Carr T. H. Davidson B. J. Hawkins H. L. (1978). Perceptual flexibility in word recognition: Strategies affect orthographic computation but not lexical access. Journal of Experimental Psychology: Human Perception and Performance, 4(4), 674–690. 10.1037/0096-1523.4.4.674 722255

[bibr9-17470218221106155] Chase C. H. Tallal P. (1990). A developmental, interactive activation model of the word superiority effect. Journal of Experimental Child Psychology, 49(3), 448–487. 10.1016/0022-0965(90)90069-K 2348161

[bibr10-17470218221106155] Coch D. Mitra P. (2010). Word and pseudoword superiority effects reflected in the ERP waveform. Brain Research, 1329, 159–174. 10.1016/j.brainres.2010.02.084 20211607 PMC2857552

[bibr11-17470218221106155] Davis M. H. (2001). Up-down staircase psychophysical method. http://www.u.arizona.edu/~kforster/dmdx/up-down_staircase.htm

[bibr12-17470218221106155] Davis M. H. Sohoglu E. (2020). Three functions of prediction error for Bayesian inference in speech perception. In Gazzaniga M. Mangun R. Poeppel D. (Eds.), The cognitive neurosciences (6th ed., pp. 177–189). MIT Press.

[bibr13-17470218221106155] Dehaene S. Cohen L. Sigman M. Vinickier F. (2005). The neural code for written words: a proposal. Trends in Cognitive Sciences, 9(7), 335–341. 10.1016/j.tics.2005.05.004 15951224

[bibr14-17470218221106155] Dienes Z. (2014). Using Bayes to get the most out of non-significant results. Frontiers in Psychology, 5, Article 781. 10.3389/fpsyg.2014.00781 PMC4114196 25120503

[bibr15-17470218221106155] Fiset D. Blais C. Ethier-Majcher C. Arguin M. Bub D. Gosselin F. (2008). Features for identification of uppercase and lowercase letters. Psychological Science, 19(11), 1161–1168. 10.1111/j.1467-9280.2008.02218.x 19076489

[bibr16-17470218221106155] Forster K. I. Forster J. C. (2003). DMDX: A Windows display program with millisecond accuracy. Behavior Research Methods, 35(1), 116–124. 10.3758/BF03195503 12723786

[bibr17-17470218221106155] Grainger J. Bouttevin S. Truc C. Bastien M. Ziegler J. (2003). Word superiority, pseudoword superiority, and learning to read: A comparison of dyslexic and normal readers. Brain and Language, 87(3), 432–440. 10.1016/S0093-934X(03)00145-7 14642545

[bibr18-17470218221106155] Grainger J. Jacobs A. M. (1994). A dual read-out model of word context effects in letter perception: Further investigations of the word superiority effect. Journal of Experimental Psychology: Human Perception and Performance, 20(6), 1158–1176. 10.1037/0096-1523.20.6.1158

[bibr19-17470218221106155] Grainger J. Jacobs A. M. (1996). Orthographic processing in visual word recognition: A multiple read-out model. Psychological Review, 103(3), 518–565. 10.1037/0033-295X.103.3.518 8759046

[bibr20-17470218221106155] Grainger J. (2018). Orthographic processing: A ‘mid-level’ vision of reading: The 44th Sir Frederic Bartlett Lecture. Quarterly Journal of Experimental Psychology, 71(2), 335–359. 10.1080/17470218.2017.1314515 28376655

[bibr21-17470218221106155] Green P. MacLeod C. J. (2016). SIMR: An R package for power analysis of generalized linear mixed models by simulation. Methods in Ecology and Evolution, 7(4), 493–498. 10.1111/2041-210X.12504

[bibr22-17470218221106155] Hannagan T. Ktori M. Chanceaux M. Grainger J. (2012). Deciphering CAPTCHAs: What a Turing test reveals about human cognition. PLOS ONE, 7(3), Article e32121. 10.1371/journal.pone.0032121 PMC3291547 22396750

[bibr23-17470218221106155] Kezilas Y. Kohnen S. McKague M. Robidoux S. Castles A. (2016). Word and pseudoword superiority effects on letter position processing in developing and skilled readers. Journal of Experimental Psychology: Human Perception and Performance, 42(12), 1989–2002. 10.1037/xhp0000273 27732042

[bibr24-17470218221106155] Kinoshita S. Kaplan L. (2008). Priming of abstract letter identities in the letter match task. Quarterly Journal of Experimental Psychology, 61(12), 1873–1885. 10.1080/17470210701781114 18609367

[bibr25-17470218221106155] Kinoshita S. Robidoux S. Mills L. Norris D. (2013). Visual similarity effects on masked priming. Memory & Cognition, 42(5), 821–833. https://doi.org/10.3758%2Fs13421-013-0388-4 10.3758/s13421-013-0388-4 PMC4055850 24343551

[bibr26-17470218221106155] Lee M. D. Wagenmakers E. J. (2014). Bayesian cognitive modeling: A practical course. Cambridge University Press.

[bibr27-17470218221106155] Lété B. Ducrot S. (2008). Visuo-attentional processing by dyslexic readers in the Reicher–Wheeler task. Current Psychology Letters, 24. http://www.cpl.revues.org/document3523.html

[bibr28-17470218221106155] Lien M. C. Allen P. Martin N. (2014). Processing visual words with numbers: Electrophysiological evidence for semantic activation. Psychonomic Bulletin & Review, 21(4), 1056–1066. 10.3758/s13423-014-0581-x 24436053

[bibr29-17470218221106155] Loftus G. R. Masson M. E. (1994). Using confidence intervals in within-subject designs. Psychonomic Bulletin & Review, 1(4), 476–490. 10.3758/BF03210951 24203555

[bibr30-17470218221106155] Marcet A. Perea M. (2017). Is nevtral NEUTRAL? Visual similarity effects in the early phases of written-word recognition. Psychonomic Bulletin & Review, 24(4), 1180–1185. 10.3758/s13423-016-1180-9 27873186

[bibr31-17470218221106155] Marcet A. Perea M. (2018a). Can I order a burger at rnacdonalds.com? Visual similarity effects of multi-letter combinations at the early stages of word recognition. Journal of Experimental Psychology: Learning, Memory, and Cognition, 44(5), 699–706. 10.1037/xlm0000477 29094993

[bibr32-17470218221106155] Marcet A. Perea M. (2018b). Visual letter similarity effects during sentence reading: Evidence from the boundary technique. Acta Psychologica, 190, 142–149. 10.1016/j.actpsy.2018.08.007 30119047

[bibr33-17470218221106155] Matuschek H. Kliegl R. Vasishth S. Baayen H. Bates D. (2017). Balancing Type I error and power in linear mixed models. Journal of Memory and Language, 94, 305–315. 10.1016/j.jml.2017.01.001

[bibr34-17470218221106155] McClelland J. L. (1979). On the time relations of mental processes: an examination of systems of processes in cascade. Psychological Review, 86(4), 287–330. 10.1037/0033-295X.86.4.287

[bibr35-17470218221106155] McClelland J. L. Rumelhart D. E. (1981). An interactive activation model of context effects in letter perception: I. An account of basic findings. Psychological Review, 88(5), 375–407. 10.1037/0033-295X.88.5.375 7058229

[bibr36-17470218221106155] Mutch J. Lowe D. G. (2008). Object class recognition and localization using sparse features with limited receptive fields. International Journal of Computer Vision, 80(1), 45–57. 10.1007/s11263-007-0118-0

[bibr37-17470218221106155] Norris D. (2006). The Bayesian reader: Explaining word recognition as an optimal Bayesian decision process. Psychological Review, 113(2), 327–357. 10.1037/0033-295x.113.2.327 16637764

[bibr38-17470218221106155] Norris D. Kinoshita S. (2012). Reading through a noisy channel: Why there’s nothing special about the perception of orthography. Psychological Review, 119(3), 517–545. https://psycnet.apa.org/doi/10.1037/a0028450 22663560 10.1037/a0028450

[bibr39-17470218221106155] Norris D. Kinoshita S. van Casteren M. (2010). A stimulus sampling theory of letter identity and order. Journal of Memory and Language, 62(3), 254–271. 10.1016/j.jml.2009.11.002

[bibr40-17470218221106155] Perea M. Duñabeitia J. A. Carreiras M. (2008). R34d1ng w0rd5 w1th numb3r5. Journal of Experimental Psychology: Human Perception and Performance, 34(1), 237–241. 10.1037/0096-1523.34.1.237 18248151

[bibr41-17470218221106155] R Core Team. (2016). R: A language and environment for statistical computing [Computer software]. R Foundation for Statistical Computing. http://www.R-project.org/

[bibr42-17470218221106155] Reicher G. M. (1969). Perceptual recognition as a function of meaningfulness of stimulus material. Journal of Experimental Psychology, 81(2), 275–280. 10.1037/h0027768 5811803

[bibr43-17470218221106155] Roettger T. (2019). Researcher degrees of freedom in phonetic research. Laboratory Phonology, 10(1), Article 1. 10.5334/labphon.147

[bibr44-17470218221106155] Rosa E. Perea M. Enneson P. (2016). The role of letter features in visual-word recognition: Evidence from a delayed segment technique. Acta Psychologica, 169, 133–142. 10.1016/j.actpsy.2016.05.016 27289422

[bibr45-17470218221106155] Rumelhart D. E. McClelland J. L. (1982). An interactive activation model of context effects in letter perception: II. The contextual enhancement effect and some tests and extensions of the model. Psychological Review, 89(1), 60–94. 10.1037/0033-295X.89.1.60 7058229

[bibr46-17470218221106155] Schubert T. M. Cohen T. Fischer-Baum S. (2020). Reading the written language environment: Learning orthographic structure from statistical regularities. Journal of Memory and Language, 114, Article 104148. 10.1016/j.jml.2020.104148

[bibr47-17470218221106155] Silvey C. Dienes Z. Wonnacott E. (2021). Bayes factors for mixed-effects models. PsyArXiv. 10.31234/osf.io/m4hju 39666535

[bibr48-17470218221106155] Simpson I. C. Mousikou P. Montoya J. M. Defior S. (2013). A letter visual-similarity matrix for Latin-based alphabets. Behavior Research Methods, 45(2), 431–439. 10.3758/s13428-012-0271-4 23055176

[bibr49-17470218221106155] Sohoglu E. Peelle J. E. Carlyon R. P. Davis M. H. (2012). Predictive top-down integration of prior knowledge during speech perception. Journal of Neuroscience, 32(25), 8443–8453. 10.1523/JNEUROSCI.5069-11.2012 22723684 PMC6620994

[bibr50-17470218221106155] Wheeler D. (1970). Processes in word recognition. Cognitive Psychology, 1(1), 59–85. 10.1016/0010-0285(70)90005-8

[bibr51-17470218221106155] Wiley R. W. Wilson C. Rapp B. (2016). The effects of alphabet and expertise on letter perception. Journal of Experimental Psychology: Human Perception and Performance, 42(8), 1186–1203. 10.1037/xhp0000213 26913778 PMC4980158

[bibr52-17470218221106155] Woolnough O. Donos C. Rollo P. S. Forseth K. J. Lakretz Y. Crone N. E. Fischer-Baum S. Dehaene S. Tandon N. (2020). Spatiotemporal dynamics of orthographic and lexical processing in the ventral visual pathway. Nature Human Behaviour, 5, 389–398. 10.1038/s41562-020-00982-w PMC10365894 33257877

